# The etiological spectrum of pediatric FUO and clinical management of PFAPA/SURF: a ten-year retrospective study

**DOI:** 10.3389/fimmu.2026.1791904

**Published:** 2026-03-26

**Authors:** Chenchen Guan, Qinhua Zhou, Jinqiao Sun, Wenjing Ying, Xiaoying Hui, Jia Hou, Lipin Liu, Chenghao Wang, Luyao Liu, Bijun Sun, Wenjie Wang, Xiaochuan Wang

**Affiliations:** 1 Department of Clinical Immunology and Allergy, Children’s Hospital of Fudan University, Shanghai, China; 2 Shanghai Institute of Infectious Disease and Biosecurity, Fudan University, Shanghai, China

**Keywords:** autoinflammatory diseases, fever of unknown origin (FUO), periodic fever aphthous stomatitis pharyngitis and cervical adenitis (PFAPA), recurrent fevers, syndrome of undifferentiated recurrent fever (SURF)

## Abstract

**Background:**

This study aimed to define the etiologies and clinical features of pediatric Fever of unknown origin (FUO), focusing on periodic fever, aphthous stomatitis, pharyngitis, and cervical adenitis (PFAPA) and the syndrome of undifferentiated recurrent fever (SURF), to guide clinical management.

**Methods:**

We retrospectively analyzed pediatric FUO cases at Fudan University Children’s Hospital (2014–2024.6), assessing clinical characteristics, immune phenotypes, laboratory parameters, and treatment responses with at least one-year follow-up.

**Results:**

Among 442 children (64.7% male), the mean age at onset was 3.42years. Infectious diseases accounted for 20.6%, mainly EBV infection. Autoinflammatory diseases were predominant (n=291), including PFAPA (n=135), and SURF (n=72). PFAPA presented earlier (mean age, 2.25 years) with regular episodes (median interval, every 25 days). SURF showed variable intervals (range, 7–120 days) and was classified as periodic (n = 38) or non-periodic (n = 34) subtypes. Isolated fever occurred in 21% of SURF periodic, whereas SURF non-periodic more often involved systemic symptoms (fatigue, headache, urticaria, and gastrointestinal complaints). During attacks, both PFAPA and SURF showed elevated CRP/ESR with neutrophilia/monocytosis and lymphopenia. Corticosteroids aborted PFAPA episodes in 90.9% but shortened afebrile intervals in 31.8%; tonsillectomy resulted in a 90.5% complete remission rate. Among non-surgical treatments, immunomodulation proved beneficial, with OM-85 yielding 45.0% complete remission and 65.0% overall clinical benefit. SURF treatment responses were heterogeneous: SURF periodic responded better to OM-85, whereas SURF non-periodic showed partial responses to colchicine or biologics.

**Conclusion:**

Autoinflammatory diseases, particularly PFAPA and SURF, were the leading causes of pediatric FUO, offering insights for management and treatment.

## Highlights

- Autoinflammatory diseases, especially PFAPA and SURF,are emerging as leading causes of pediatric FUO.
- Accurate differentiation between PFAPA and SURF requires careful consideration of fever patterns and systemic features.
- OM-85 and tonsillectomy provide effective long-term remission in PFAPA, while SURF treatment requires more flexibility.

## 1 Introduction

Fever is a physiological response triggered by the body’s thermoregulatory system to maintain internal homeostasis, typically defined as an elevation in core body temperature above normal values (≥37.9 °C) (1, 2). Fever of unknown origin (FUO) was first introduced by Petersdorf and Beeson in 1961in adults as a fever >38.3 °C (101°F) lasting at least three weeks, with no established diagnosis after one week of hospital-based evaluation (3). Pediatric FUO is typically defined as a fever lasting for ≥1 to 2 weeks (prolonged fever), with a body temperature >38.0 °C on most days, and without a clear etiology despite thorough clinical assessment and basic laboratory workup (4–6). Defining FUO solely by prolonged fever no longer meets clinical needs. When an individual experiences three or more episodes of unexplained fever within a 6-month period, with recurrences separated by intervals of at least 7 days, this pattern is termed recurrent fever (2, 7), both recurrent fever and the traditional prolonged fever can be broadly described as FUO (8).

Investigating the etiology of FUO is central to clinical diagnosis and management. FUO is typically categorized into infectious or non-infectious, including autoimmune diseases, autoinflammatory diseases, malignancies, inherited metabolic disorders (such as adrenal insufficiency, hyperthyroidism), and thermoregulatory dysfunctions such as central fever due to the elevation of the body temperature set point, such as brain hemorrhage or central tumors. Additional categories include drug-induced fever, miscellaneous syndromes, and truly undiagnosed cases (5, 9). Previous studies have shown that infectious etiologies are the most common causes of fever, followed by autoimmune diseases and malignancies (6, 9–15). Recently, with advancements in diagnostic technologies and increasing awareness of non-infectious etiologies, and the broadening of fever-pattern classifications, the distribution of FUO causes has shifted, in particular, the proportion of autoinflammatory and undiagnosed conditions has steadily increased. In a 15-year single-center pediatric FUO cohort (n=100), infectious causes were only 19%, while non-infectious and undiagnosed etiologies predominated (4), up to 50% of patients with fever of unknown origin (FUO) remain undiagnosed despite extensive evaluation (16). Autoinflammatory syndromes as causes of fever of unknown origin (17).

Systemic autoinflammatory diseases (SAIDs) are disorders resulting from dysregulation of the innate immune system. To date, more than 40 monogenic entities have been described (18). When genetic testing is negative and infections, malignancies, and autoimmune diseases have been excluded, a subset of patients present with unexplained recurrent fevers. A common phenotype is PFAPA syndrome, a multifactorial disorder and the most prevalent periodic fever syndrome (19). Some patients do not fulfill conventional PFAPA criteria has been termed as the SURF (20). Data from European autoinflammatory registries reveal substantial phenotypic overlap between PFAPA and SURF, highlighting the need for more granular evaluation and clearer delineation of these entities (21). Further research and detailed characterization of PFAPA and SURF are needed to refine disease definitions and classification.

To better characterize the etiological spectrum and clinical features of FUO, we conducted a retrospective cohort study of children diagnosed with FUO in the Department of Clinical Immunology and Allergy, Fudan University Children’s Hospital, over a 10-year period (2014–2024.6). Standardized case definitions were used to differentiate prolonged and recurrent fever and applied these definitions consistently to a real-world cohort followed for at least 12 months, with a specific focus on PFAPA and SURF, providing actionable guidance for referral and management pathways.

## 2 Methods

### 2.1 Selection criteria of FUO

Prolonged fever defined as a rectal temperature ≥38.0 °C or axillary temperature ≥37.3 °C lasting for more than two weeks, and if no definitive diagnosis could be established after routine evaluations (22). Recurrent fever occurs frequently and is defined as at least three episodes of unexplained fever within six months, with intervals exceeding seven days (2, 7) We enrolled FUO patients presenting with prolonged and recurrent fever (at least 6 episodes) referred to our hospital between 2014 and June 2024.

### 2.2 Diseases group

Cases were grouped as infectious, autoimmune, autoinflammatory, neoplastic, abnormal thermoregulation, and undiagnosed; categories were applied uniformly, follow-up was ≥12 months, and timelines used discharge as the endpoint. Pseudo fever, drug fever, and non-autoinflammatory inborn errors of immunity (IEI) were excluded.

PFAPA syndrome was diagnosed according to the modified Marshall criteria and the Eurofever/PRINTO classification framework (23, 24). Patients were required to meet either of the following: (1) onset before age 5, with periodic fevers and at least one of the following symptoms, aphthous stomatitis, pharyngitis/tonsillitis, or cervical lymphadenitis, with no systemic inflammation between episodes (CRP <0.2 mg/L; ESR <15 mm/h); or (2) at least seven of the following eight features: pharyngitis/tonsillitis, cervical lymphadenitis, fever lasting 3–6 days, and regular periodicity; absence of diarrhea, chest pain, rash, and arthritis. Monogenic autoinflammatory syndromes (eg: familial Mediterranean fever (FMF), Hyper-IgD syndrome (HIDS), TNF receptor associated periodic syndrome (TRAPS), cryopyrin associated periodic syndrome (CAPS)), cyclic neutropenia, immunodeficiency or autoimmunity were excluded based on clinical criteria.

**Figure 1:**
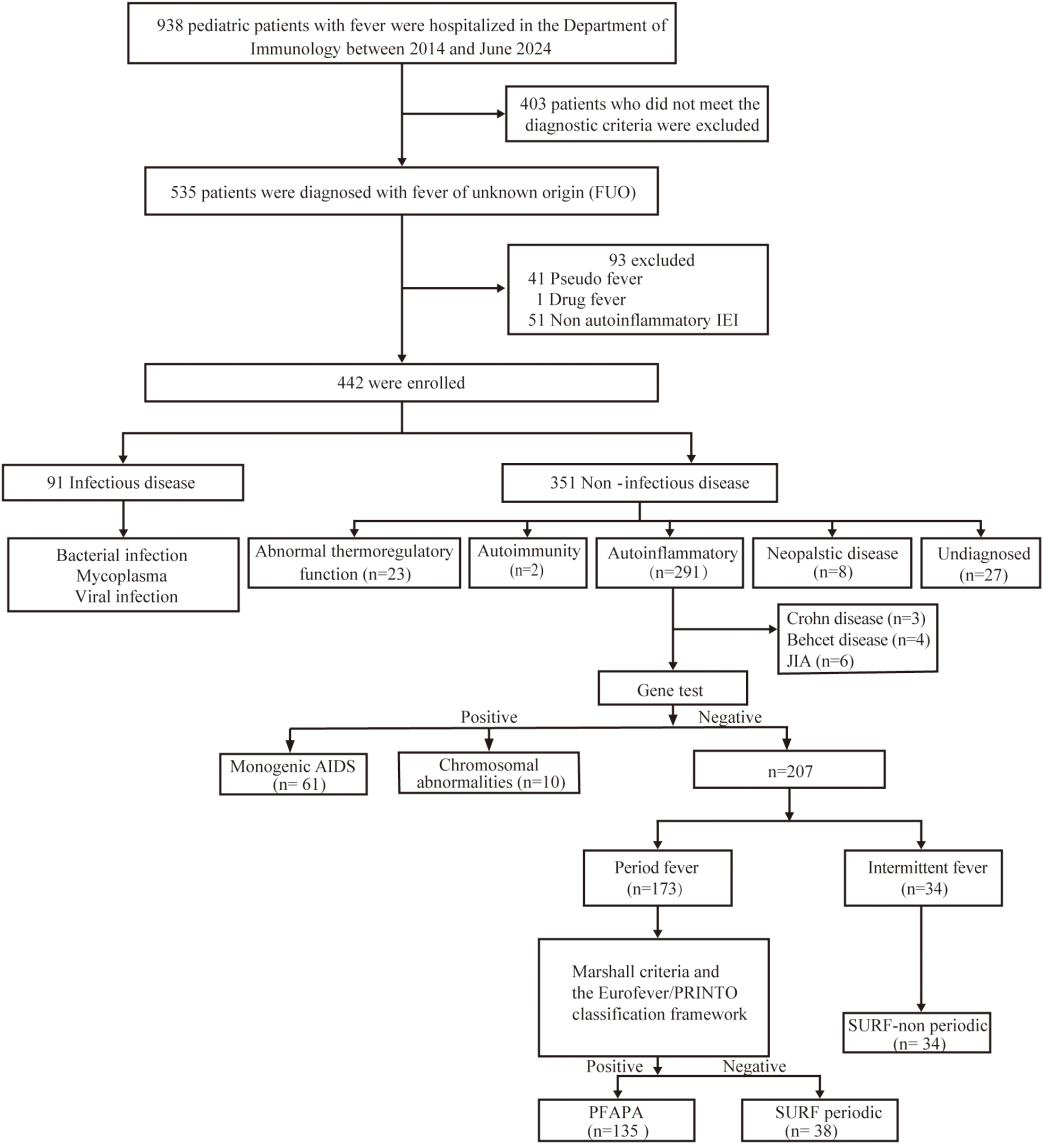
Study design and patient selection flowchart. Flowchart of screening and grouping of 442 pediatric patients with fever of unknown origin (FUO) treated in the Department of Clinical Immunology and Allergy, Fudan University Children’s Hospital between 2014 and June 2024.

The criteria for SURF included intermittent systemic inflammation with normalization between episodes, normal growth and development, and clinical exclusion of PFAPA, monogenic autoinflammatory diseases, cyclic neutropenia, immunodeficiencies, and autoimmune disorders. The study design is shown in **Figure 1**.

The study was approved by the Ethics Committee of the Children’s Hospital of Fudan University. Written informed consent was obtained from the parents or legal guardians of all participants.

### 2.3 Immunological evaluation

Routine blood counts and inflammatory markers include C-reactive protein, erythrocyte sedimentation rate, and serum amyloid A were performed. Based on temperature and complete blood count results measured on the day of admission, cases were classified into acute attacks or remission period. Serum immunoglobulins, such as IgG, IgA, IgM and IgE, were detected using nephelometry. The distribution of lymphocyte subsets was measured through flow cytometry (Becton Dickinson, Franklin Lakes, NJ, USA). In this procedure, we employed validated antibodies, including anti-CD3 (UCHT1), anti-CD8 (RPAT8), anti-CD4 (RPA-T4), anti-CD19 (HIB19), anti-CD16 (3G8), anti-CD56 (QA17A16) (all from BD Biosciences). Cytokine was determined in supernatants using commercial ELISA kits for human TNF-a (Biolegend.430204), human IL-6 (Biolegend,430504). After exclusion of infectious, neoplastic, and autoimmune conditions, genetic testing was performed in patients with suspected autoinflammatory disease.

### 2.4 Statistical analysis

Categorical variables were summarized as frequencies and percentages, while numeric variables were presented as mean (SD) or median (IQR). *t*-test was used to determine the differences of inflammatory mediators between acute attacks and remission period. Group comparisons of categorical variables were performed using the chi-square test. *P*≤ 0.05 was considered statistically significant. All analyses were performed using SPSS 21.

## 3 Result

### 3.1 General characteristics of our cohort

Between 2014 and June 2024, 442 pediatric patients diagnosed with FUO were enrolled, comprising 64.7% males and 35.3% females. The mean age of onset was 3.42 years with a fever duration ranging from 2 weeks to 8 years, and an average duration of 2 years.

### 3.2 Etiological distribution

#### 3.2.1 Infectious disease

**Figure 2:**
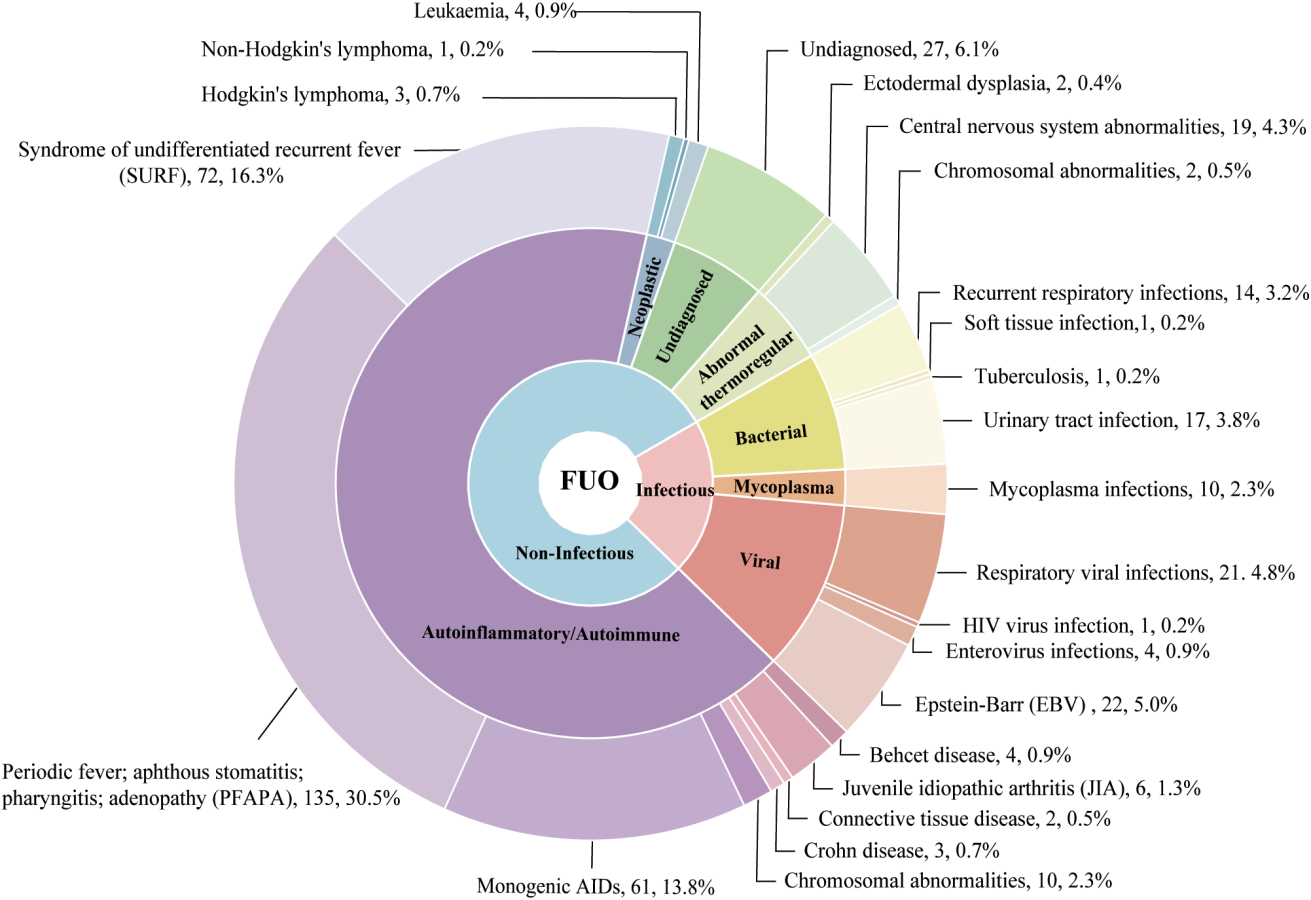
Etiologic distribution of pediatric fever of unknown origin. Etiologic composition of 442 children with fever of unknown origin (FUO) evaluated in the Department of Clinical Immunology and Allergy, Fudan University Children’s Hospital, 2014–2024.6. Autoinflammatory diseases were most frequent (65.8%, n=291), followed by infectious diseases (20.6%, n=91), thermoregulatory disorders (5.2%, n=23), neoplasms (1.8%, n=8), and undiagnosed cases (6.1%, n=27), autoimmune disease(0.5%, n=2). The pie chart displays the relative proportions of each category.

In our cohort, infectious diseases accounted for 20.6% (n = 91), with a median age of 2.0 years (IQR, 0.52–6.87), and 58.2% of cases occurred in children under 3 years of age. 49.4% of patients presenting with temperatures ranging from 39.1 °C to 41 °C (**Table 1**). The most common infection was EBV (n=22, 5%) (**Figure 2**), with the mean age of 5.4 years. Urinary tract infections (UTIs) were identified in 17 patients (3.8%), including 5 females and 12 males, with a median age of 1.16 years and a median peak temperature of 39.1 °C. Notably, 52.9% (9/17) of cases were attributable to vesicoureteral reflux, six patients underwent surgical treatment. Gram-negative bacteria were the most common pathogens in UTIs. (52.9%, 9/17).

**Table 1:** Baseline demographics and fever features of 442 children with FUO.

Characteristic	Infectious diseases (n=91)	Neoplastic diseases (n=8)	Abnormal thermoregulatory function (n=23)	Autoimmune/Autoinflammatory diseases (n=293)	Undiagnosed (n=27)
Male: Female	68:23	8:0	14:9	181:112	13:14
Age of onset					
0-1y	33 (36.26%)	1 (12.50%)	20 (86.95%)	112 (38.23%)	1 (3.70%)
1-3y	20 (21.98%)	1 (12.50%)	3 (13.05%)	95 (32.42%)	2 (7.41%)
3-6y	15 (16.48%)	3 (37.50%)	0	57 (19.45%)	5 (18.52%)
6-12y	18 (19.78%)	3 (37.50%)	0	22 (7.51%)	12 (44.44%)
>12y	5 (5.50%)	0	0	7 (2.39%)	7 (25.93%)
Age of onset, median (IQR), years	2.00 (0.52, 6.87)	4.96 (2.67,7.89)	0.50 (0.33,0.83)	2.00 (0.92, 4.00)	9.00 (5.08, 11.12)
Peak fever					
37.3-38 °C	9 (9.89%)	0	14 (60.87%)	7 (2.39%)	4 (14.82%)
38.1-39 °C	37 (40.66%)	4 (50.00%)	4 (17.39%)	91 (31.06%)	14 (51.85%)
39.1-41 °C	45 (49.45%)	4 (50.00%)	5 (21.74%)	190 (64.85%)	6 (22.22%)
>41 °C	0	0	0	5 (1.70%)	3 (11.11%)
Peak fever, median (IQR), °C	39.1 (38.7,39.7)	39.2 (38.5, 39.5)	38.0 (37.5, 39.0)	39.5 (39.0, 40.0)	38.7 (38.0, 39.9)

#### 3.2.2 Neoplastic diseases

Neoplastic diseases accounted for 1.8% (n = 8) of the cohort, including 1 case of non-Hodgkin’s lymphoma, 3 cases of Hodgkin’s lymphoma, and 4 cases of leukemia (**Figure 2**). The median age of these patients was 4.96 years and a median peak temperature of 39.2 °C (**Table 1**).

#### 3.2.3 Abnormal thermoregulation

Abnormal thermoregulation, including centric fever and ectodermal dysplasia, accounted for 5.2% (n = 23) of enrolled cases (**Figure 2**), with a median age of 0.5 years (IQR, 0.33–0.83), and 87% of these patients were under the age of 1. The majority of cases presented with low-grade fever, with 60.9% of patients having temperatures below 38 °C (**Table 1**).

#### 3.2.4 Autoimmune disease

Two patients (0.5%) were diagnosed with undifferentiated connective tissue disease, one 11 years old male and one 10 years old female. One patient presented with recurrent fever for 9 months, while the other had persistent fever for nearly one month.

#### 3.2.5 Undiagnosed

The undiagnosed group (n = 27) accounted for 6.1% of cases (**Figure 2**). This group had the oldest median age (9 years), with 44.5% of patients aged between 6 and 12 years and 25.9% over the age of 12. These patients typically presented with moderate fevers (51.9% had temperatures between 38.1 °C and 39 °C), but this group had the highest proportion of hyperpyrexia, with 11.1% of patients experiencing temperatures greater than 41 °C (**Table 1**).

### 3.3 Autoinflammatory disease

#### 3.3.1 General characteristics

Among 291 FUO patients diagnosed with autoinflammatory diseases, genetic testing identified chromosomal abnormalities in 10 cases, which were published before (25), and monogenic autoinflammatory diseases in 61 cases, including *NLRP3, NLRP12, MVK, and MEFV.* These patients typically exhibited an earlier age at onset and more frequent conjunctivitis, arthralgia, rash, and abdominal pain during febrile episodes (with portions of these findings reported previously) (26). 135 patients with recurrent fever initially classified as FUO were ultimately diagnosed with PFAPA, consisting of 82 males (60.7%) and 53 females (39.3%). Another 72 patients did not meet PFAPA criteria and were categorized as having the SURF, comprising 49 males (68.1%) and 23 females (31.9%) (**Figure 2**).

#### 3.3.2 PFAPA and SURF

##### 3.3.2.1 General characteristics

Autoinflammatory patients showed a remarkably early age of onset: 70.6% of patients developed symptoms before 3 years of age and 90.1% before 6 years (**Table 1**). High-grade fever was the predominant presentation (64.8%). As shown in **Table 2**, to further explore the clinical characteristics of the disease, the SURF cohort was subdivided based on fever pattern into two groups: SURF periodic (n = 38; 24 males, 14 females), characterized by relatively regular intervals between episodes, and SURF non-periodic (n =34; 25 males, 9 females), with no discernible periodicity. Demographic characteristics and febrile episode feature of all groups are summarized in **Table 2**. Comparative analyses among the three groups demonstrated no statistically significant differences in sex, onset age, peak temperature, or fever duration. PFAPA and SURF periodic showed regular periodic fevers, with median inter-episode intervals of 25 days and 30 days, respectively, the frequency of episodes in SURF non-periodic patients was irregular (range: 7–120 days).

**Table 2:** Distinguishing clinical characteristics recurrent fever patients.

Variables	PFAPA	SURF periodic	SURF non-periodic	*P* value
Patients, n	135	38	34	
Male: Female	82:53	24:14	25:9	n.s.
Age of onset, median (IQR), years	1.50 (1.00, 3.08)	1.75 (0.64, 3.29))	2.00 (0.92, 4.63)	n.s.
Tmax, median (IQR),°C	39.7 (39.0, 40.0)	40.0 (39.0, 40.0)	39.5 (39.0, 40.0)	n.s.
Episode duration, median (IQR), days	4.0 (3.5, 5.0)	4.0 (3.5, 5.0)	4.0 (3.0, 5.0)	n.s.
Asymptomatic interval, median (IQR), days	25 (20, 30)	30 (19, 30)	30 (14, 45)	<0.001
Family history of recurrent fever (%)	28.9	5.3	23.5	<0.01
Fever only (%)	0	21.0	0	<0.001
Pharyngitis (%)	28.9	5.3	17.6	0.007
lymphadenopathy (%)	64.4	34.2	67.6	0.002
Aphthous ulcers (%)	31.9	7.9	32.3	0.011
Tonsillitis (%)	91.9	7.9	52.9	<0.001
Maculopapular rash (%)	3.0	5.3	5.9	n.s.
Urticaria (%)	17.8	34.2	44.1	0.001
Abdominal pain (%)	20.0	38.5	32.3	0.03
Vomiting (%)	9.6	13.1	20.6	n.s.
Diarrhea (%)	7.4	7.9	26.5	0.008
Myalgia (%)	3.7	18.4	11.8	0.007
Arthritis (%)	11.1	18.4	20.6	n.s.
Arthralgia (%)	11.9	13.2	26.5	n.s.
Headache (%)	14.8	15.8	35.3	0.02
Febrile convulsion (%)	17.0	5.2	8.8	n.s.

##### 3.3.2.2 Clinical characterization

As shown in **Table 2**, A family history of recurrent fever (one or both parents exhibiting similar recurrent fever episodes during their childhood) was more frequently observed in the PFAPA group (28.9%) compared with the SURF periodic (5.3%) and SURF non-periodic groups (23.5%). During febrile episodes, Tonsillitis was most prevalent in the PFAPA (91.9%). Cutaneous rashes were more common in the SURF groups (34.2% in SURF periodic and 44.1% in SURF non-periodic) compared with PFAPA (17.8%). SURF periodic and SURF non-periodic subtype demonstrated distinct clinical profiles. In the SURF periodic group, lymphadenopathy (34.2%) and aphthous ulcers (7.9%) were least frequent, whereas abdominal pain (38.5%) and myalgia (18.4%) were most frequent. 21% of patients present with isolated fever. In the SURF non-periodic group, characterized by irregular fever cycles, systemic autoinflammatory manifestations involvement was particularly prominent. Fatigue (33.3%) and headache (35.3%) frequently preceded febrile episodes were most common across the three groups. Moreover, 44.1% of patients in this group developed cutaneous rashes. Gastrointestinal symptoms were more common, including abdominal pain (32.3%), vomiting (20.6%), and diarrhea (26.5%). Additionally, sinusitis was reported in 27% of these patients. Additionally, the inflammatory symptoms during each episode were inconsistent and difficult to predict.

##### 3.3.2.3 Laboratory results and immunological profiles

We examined the inflammatory characteristics and immune function of patients from different subgroups during both acute attacks and remission periods. During acute attacks, all subgroups exhibited elevated levels of CRP and ESR, with increased white blood cells, neutrophils, and monocytes, alongside reduced lymphocyte counts (**Supplementary Figure 1**). The SURF periodic group had higher white blood cell (12.97 ± 4.06 × 10^9^/L) and monocyte levels (1.30 ± 0.6 × 10^9^/L) compared to others, but these differences were not statistically significant. Flow cytometric analysis showed that although the absolute counts of CD3^+^, CD4^+^, CD19^+^, and natural killer (NK) cells were within reference ranges (27), immunodisruption is present regardless of disease remission or flare, characterized by an imbalance in the CD4/CD8 ratio. 46.1% of patients in the SURF periodic group exhibited an increase in CD19^+^ cells, while 75.0% of patients in the SURF non-periodic group showed a decrease in CD19^+^ cells (**Supplementary Table 1**). In addition, we compared inflammatory markers between autoinflammatory and infectious diseases, no statistically significant differences were found (**Supplementary Table 2**). Disease flares were associated with increased IL-6 and TNF-α levels. For example, IL-6 levels in PFAPA, SURF periodic, and SURF non-periodic were 20.15-114.52 (IQR), 11.8-59.3 (IQR), and 5.7-91.1(IQR), respectively (**Supplementary Table 1**).

##### 3.3.2.4 Treatment and outcomes

Among children diagnosed with PFAPA, 44 (32.6%) received corticosteroids (e.g. prednisone or methylprednisolone) during febrile episodes. Of these, 90.9% achieved defervescence and clinical remission within 24 hours, though 31.8% experienced a shortened afebrile interval and increased attack frequency. In the SURF-period group, 10 patients received corticosteroids, with 7 (70.0%) achieved defervescence within 24 hours and 1 (10.0%) experienced a shortened afebrile interval. In the SURF-non periodic group, 13 patients received corticosteroids, with 9 (69.2%) achieving defervescence within 24 hours and 2 (15.4%) experiencing a shortened afebrile interval (**Supplementary Table 3**).

**Figure 3:**
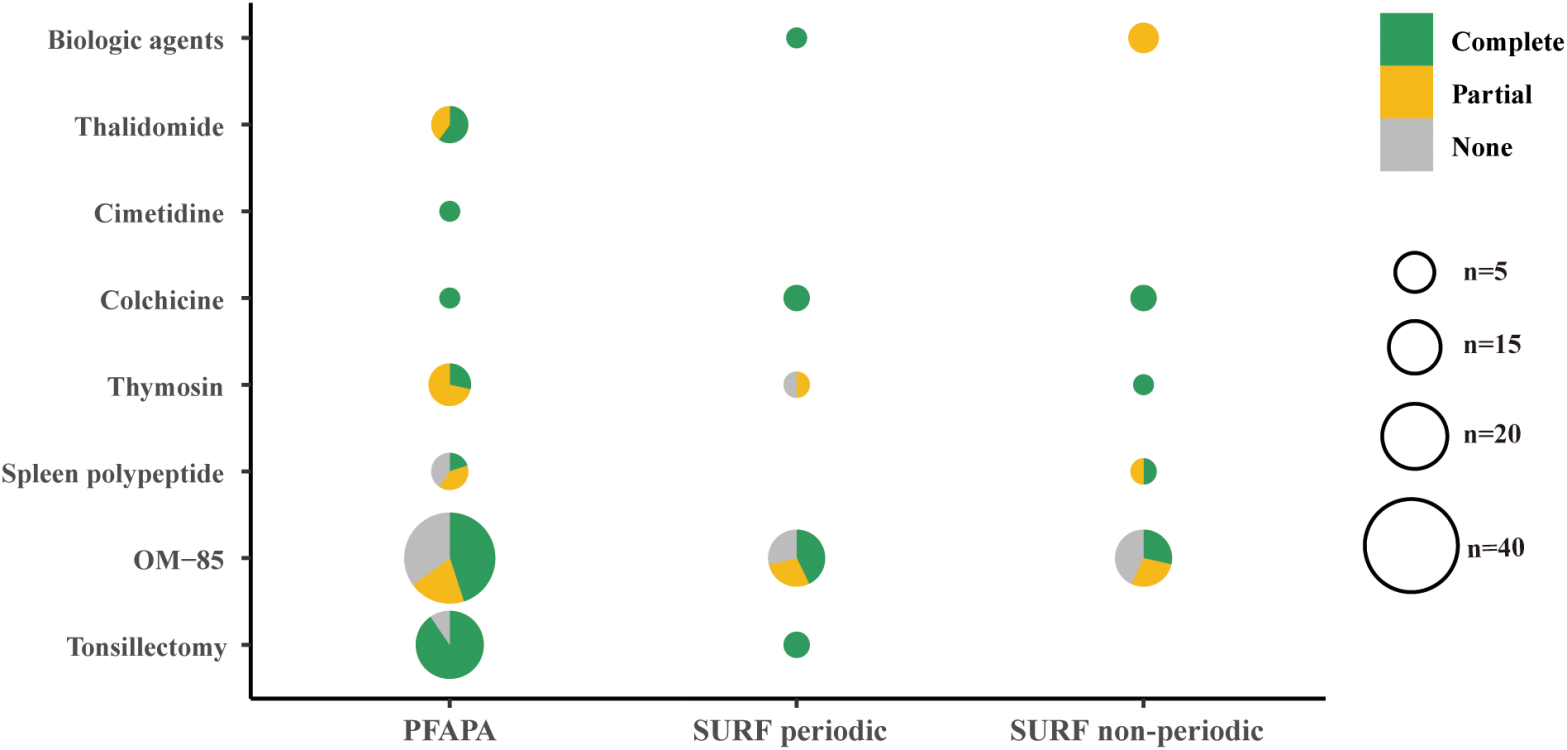
Treatments used and their responses in patients. Showed remission outcomes during follow-up among different patient groups treated with various therapeutic modalities. Complete remission: the disappearance of all clinical symptoms and the return of CRP levels and the ESR to normal values during the follow-up period. Partial remission: not meeting the criteria for complete remission but exhibiting a reduction of at least 50% in the average number of fever episodes per year and/or the absence of persistent inflammatory disease during follow-up. None: no response.

We followed 99 PFAPA patients (median follow-up of 3.5 years; IQR 2.0–5.5 years) via telephone or outpatient clinics from 2018 to June 2024. As shown in **Figure 3**, at discharge, a subset of PFAPA patients initiated immunomodulatory therapy (OM-85, spleen polypeptide, or thymosin). OM-85 among recipients (n = 40; 40.4%), the complete remission (CR) rate within three months after completing therapy was 45.0%, and the overall clinical benefit (CR + partial remission [PR]) was 65.0%; by comparison, the thymosin group (n = 7) achieved CR and PR rates of 28.6% and 71.4%, respectively, whereas the spleen polypeptide group (n = 5) had CR (20%) and PR (40%). During follow-up, a small number of patients received long-term anti-inflammatory therapy, colchicine (n = 1), cimetidine (n = 1), or thalidomide (n = 5). In addition, 21 patients underwent tonsillectomy; 19 of whom maintained sustained remission (90.5% CR) after a median follow-up of 12 months (**Supplementary Table 3**).

SURF treatment responses were more heterogeneous. In the SURF periodic subgroup (21 patients with evaluable follow-up; median follow-up, 4 years; IQR, 2–5 years), 14 patients (66.7%) received OM-85, of whom 10 (71.4%) achieved complete or partial remission within three months after completing therapy. Two patients (9.5%) received long-term colchicine and achieved complete remission; two (9.5%) underwent tonsillectomy with clinical remission; two (9.5%) opted for traditional Chinese medicine and achieved complete remission; Three patients (14.3%) experienced spontaneous remission. In the SURF non-periodic subgroup (23 patients with evaluable follow-up; median follow-up, 2.7 years; IQR, 2.0–3.0), 14 patients (60.9%) received OM-85, yielding complete remission in 4 (28.6%), partial remission in 4 (28.6%). Additionally, 3 patients (13.0%) received biologic agents with partial remission, 2 patients (8.7%) received regular corticosteroids with partial remission, 2 patients (8.7%) received colchicine with complete remission, and 2 (8.7%) had spontaneous remission (**Supplementary Table 3**).

## 4 Discussion

Fever of unknown origin (FUO) remains a diagnostic challenge. Traditionally, infectious etiologies are the most common causes of fever, followed by connective tissue diseases and malignancies (6, 10, 28). In current clinical pathways, many infections are identified and managed earlier in emergency/general pediatrics or infectious disease services, facilitated by standard microbiological testing and point-of-care diagnostics (POCT) (29). Moreover, the increasing availability of molecular diagnostics, particularly metagenomic next-generation sequencing (mNGS), has enabled more rapid and accurate detection of previously difficult-to-diagnose pathogens, further altering the diagnostic spectrum observed in referred FUO cohorts. In our cohort, infectious diseases accounted for 20.6%. What’s more, as Department of Clinical Immunology and Allergy, our cohort may be enriched for immune-mediated/autoinflammatory fever cases, particularly PFAPA and SURF. This study delves into these conditions, providing guidance for referral and management.

PFAPA is regarded as a complex genetic disorder driven by polygenic susceptibility, environmental factors, and developmental influences (30). It is characterized by mucosal/tonsillar-predominant, periodic flares without aberrant pyrin inflammasome activation. In contrast, SURF involves systemic symptoms, irregular periodicity, and TcdA-induced pyrin inflammasome activation, reversible by colchicine (31), but retain an IL-1 signature (32–34). In our cohort, consistent with prior reports that 25–50% of patients have first-degree relatives with recurrent fever (35). PFAPA family history was present in 28.9%, exceeding that observed in SURF. Prominent gastrointestinal involvement has been reported in SURF and may help distinguish it from PFAPA (32) SURF patients are more likely to develop rashes and joint pain (36) at least 40% of patients experience fatigue during febrile episodes (21). Our data further reveal heterogeneity within SURF: SURF periodic subgroup in which fever often co-occurs with myalgia and SURF non periodic subgroup characterized by irregular fever cycles and broader multisystem involvement, typically including gastrointestinal symptoms, cutaneous rashes, and prodromal fatigue and headache.

Laboratory analyses during febrile episodes revealed elevated inflammatory markers (CRP, ESR) and neutrophilia across all subgroups, supported previous research (37) During febrile episodes, Prior studies indicate that SURF patients exhibited higher levels of pro-inflammatory cytokines, including IL-1β, IL-6, IL-8, and IL-17A (36). In our cohort, IL-6 and TNF-α were markedly elevated, no significant differences were observed. Both PFAPA and SURF patients exhibit marked yet non-specific systemic inflammatory responses during febrile episodes, and routine laboratory parameters as well as peripheral cytokine profiles are insufficient to reliably distinguish between the two conditions. Moreover, persistent tonsillar inflammation and dysbiosis of the tonsillar microbiota are evident even between flares. Tejesvi et al. (38) reported altered tonsillar microbiota in PFAPA, with increased Cyanobacteria (microcystin producers) and reduced Streptococcus compared with healthy controls. These findings underscore the need for more comprehensive multi-omics approaches and in-depth mechanistic studies to delineate the differences between PFAPA and SURF and to identify robust discriminative biomarkers. In our cohort, routine inflammatory markers (CRP, ESR, and WBC) showed substantial overlap between infectious and autoinflammatory cases and therefore did not reliably distinguish periodic fever from infection. Accordingly, differentiation relied more on the overall clinical pattern (e.g., episodic course and associated features), targeted microbiologic testing when indicated, and longitudinal follow-up.

Therapeutically, the therapeutic goal in PFAPA is to reduce attack severity and frequency or achieve remission. Acetaminophen/NSAIDs provide symptomatic antipyresis; corticosteroids rapidly abort episodes but do not prevent recurrence and may increase attack frequency (39, 40). IL-1 receptor antagonists (e.g, anakinra) show short-term benefit, but evidence is limited to small, uncontrolled studies (41). Evidence for cimetidine and montelukast remains insufficient (42, 43); colchicine may prolong afebrile intervals yet rarely induces stable remission and may be more suitable for children with prominent aphthous stomatitis (44, 45). Tonsillectomy is a potential option, but existing randomized studies are small and heterogeneous (46); What’s more, owing to its invasive nature, tonsillectomy is not routinely recommended. SURF patients respond well to colchicine, possibly owing to involvement of the pyrin inflammasome in SURF pathophysiology (31). Interestingly, dysregulation of inflammatory cytokines and immune homeostasis is considered a key potential mechanism underlying disease flares, immunomodulation may be effective for recurrent fever. Study shows that the use of Streptococcus salivarius K12 (SS K12) or probiotics may help reduce fever episodes associated with PFAPA and related symptoms (47–49). Thirty-seven children clinically diagnosed with PFAPA received combined pidotimod and bacterial lysate therapy; at 1 year, 67.5% (25/37) achieved complete remission, and 10.8% (4/37) remained in complete remission at the end of year-2 follow-up (50). In our cohort, demonstrates phenotype dependent therapeutic heterogeneity. In the acute phase, corticosteroids rapidly abort attacks; in PFAPA, defervescence is faster but carries a higher risk of afebrile-interval shortening than in SURF, supporting their use as rescue rather than maintenance therapy. For prevention and maintenance, immunomodulation confers clinical benefit; among available modalities, OM-85, an oral bacterial lysate, has extensively validated safety and efficacy and is thus more acceptable to clinicians and patients. Tonsillectomy achieved high and durable remission rates and is often used as a definitive strategy. A small number of cases attained complete remission while receiving colchicine, cimetidine, or thalidomide, suggesting that anti-inflammatory agents can serve as selective, risk-controlled adjuncts. Although colchicine may prolong afebrile intervals, in real-world settings, its broader uptake may also be constrained by gastrointestinal adverse effects and the need for daily long-term dosing and monitoring, and many parents in our cohort favored OM-85 as an immunomodulatory option, which likely contributed to the low utilization of colchicine. Responses in SURF were more heterogeneous: SURF-periodic behaved more “PFAPA-like,” with relatively better responses to OM-85, whereas the SURF non-periodic exhibited attenuated responses to immunomodulation, some benefit from colchicine, and occasional need for biologics. Collectively, these observations support a phenotype and prior response guided stepwise strategy: intermittent corticosteroids for acute rescue; in PFAPA, prioritize OM-85 or tonsillectomy; and in SURF particularly SURF non-periodic employ more flexible combinations of pharmacologic and non-pharmacologic modalities. At present, there is a lack of expert consensus and standardized clinical pathways for the management of such children with recurrent fever. Multicenter collaborative studies are urgently needed to refine clinical practice and to strengthen the evidence base.

### 4.1 Limitations of the study

This study has several limitations. First, it is a single-center retrospective study, which may introduce selection bias. Furthermore, the classification criteria for PFAPA and SURF are still evolving, and the diagnostic ambiguity in clinical practice may affect the categorization of certain cases. Future research should involve multi-center collaboration and further employ genomics and big data analysis to more accurately identify the different types of recurrent fevers.

## 5 Conclusion

In conclusion, autoinflammatory diseases most notably PFAPA and SURF have emerged as the predominant etiologies of pediatric FUO in our cohort. Despite substantial overlap in clinical presentation, these conditions are phenotypically heterogeneous requiring careful differentiation based on fever periodicity and systemic features. Future multicenter investigations incorporating multi-omics approaches are warranted to clarify disease mechanisms and to support the development of standardized diagnostic algorithms and treatment pathways.

## Data Availability

The original contributions presented in the study are included in the article/**Supplementary Material**. Further inquiries can be directed to the corresponding authors.

## Supplementary material

Supplementary Figure 1 Inflammatory phenotype of patients. **(a–e)**: WBC **(a)**, Neutrophil **(b)**, lymphocyte **(c)**, monocyte **(d)** levels during acute attacks (AA) and the remission period (RP). * *P < 0.05*, ***P < 0.01*, *** *P < 0.001*.
