# Supplementary figures and images for: The etiological spectrum of pediatric FUO and clinical management of PFAPA/SURF: a ten-year retrospective study
Source: Front Immunol. 2026 Mar 26;17:1791904. doi: 10.3389/fimmu.2026.1791904 (PMC13062182; doi:10.3389/fimmu.2026.1791904)

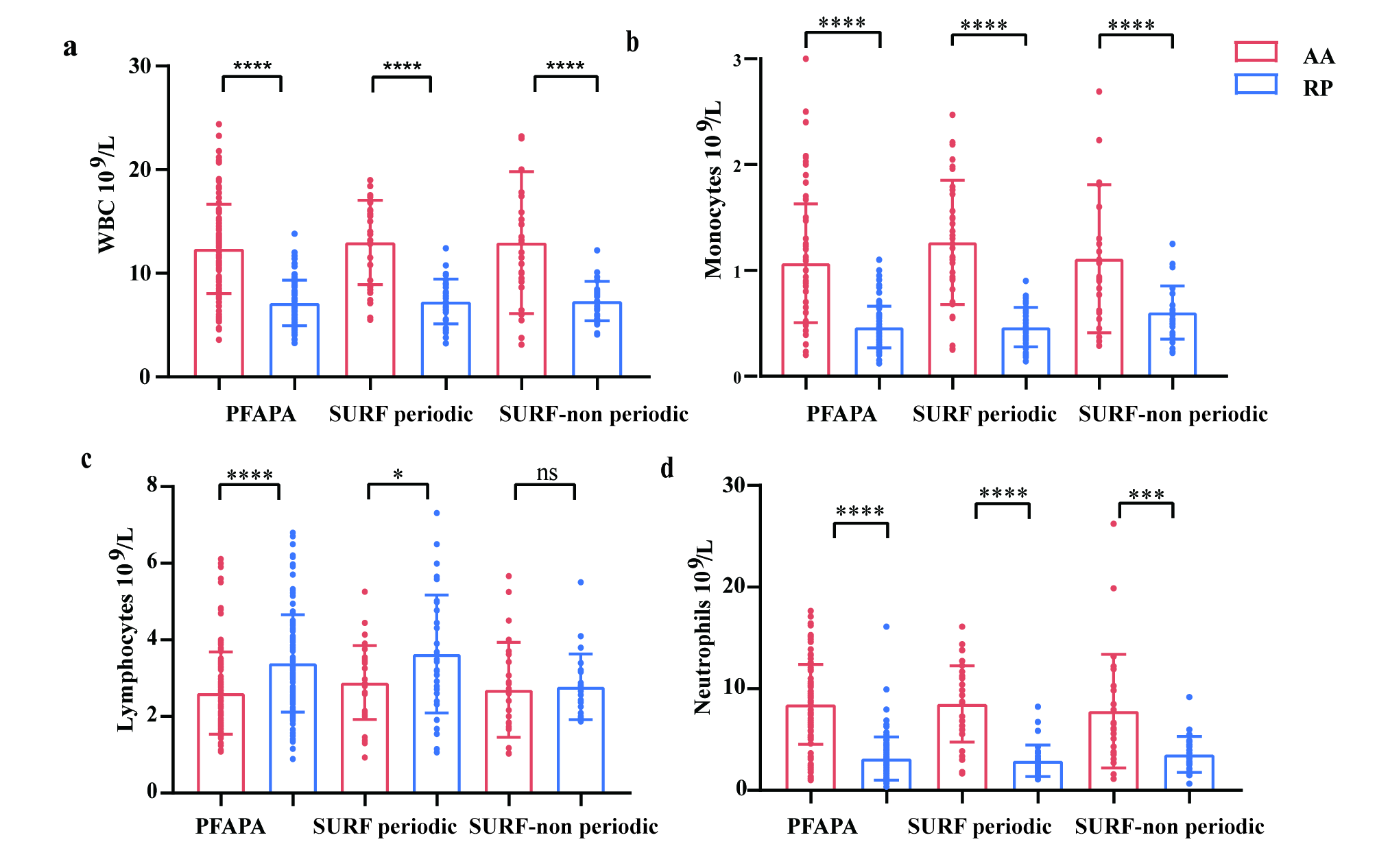

Supplement: Supplementary Figure 1 — Inflammatory phenotype of patients. (a–e): WBC (a), Neutrophil (b), lymphocyte (c), monocyte (d) levels during acute attacks (AA) and the remission period (RP). * P < 0.05, **P < 0.01, *** P < 0.001. [file Image1.tif]
